# Effectiveness of a team-level participatory approach aimed at improving sustainable employability among long-term care workers: a randomized controlled trial

**DOI:** 10.5271/sjweh.4201

**Published:** 2025-05-01

**Authors:** Ceciel H Heijkants, Madelon LM van Hooff, Astrid de Wind, Sabine AE Geurts, Prof Dr, Cécile RL Boot

**Affiliations:** 1 Radboud University, Behavioural Science Institute, The Netherlands.; 2 Open Universiteit, Faculty of Psychology, Heerlen, The Netherlands.; 3 Amsterdam UMC location University of Amsterdam, Public and Occupational Health, Amsterdam, The Netherlands.; 4 Amsterdam Public Health research institute, Societal Participation and Health, Amsterdam, The Netherlands.; 5 Amsterdam UMC, VU University, department of Public and Occupational Health, Amsterdam, The Netherlands.

**Keywords:** autonomy, basic psychological need, competence, need for recovery, participatory workplace intervention, relatedness, self-managing team

## Abstract

**Objectives:**

This study aimed to evaluate one-year effects of a team-level participatory workplace intervention on need for recovery and satisfaction of the needs for autonomy, competence and relatedness among long-term care workers by means of a randomized controlled trial.

**Methods:**

Teams of long-term care workers were randomly assigned to the intervention group (ten teams; N=78) or the wait-list control group (ten teams; N=58). The intervention consisted of a problem inventory, related to the needs for autonomy, competence and relatedness, a brainstorm towards solutions and an action plan divided over three meetings guided by a facilitator. The primary outcome was need for recovery and secondary outcomes were the satisfaction of the needs for autonomy, competence and relatedness. Outcomes were measured at baseline and after 6, 9 and 12 months. Linear mixed model analyses were performed in R.

**Results:**

There was no significant difference in need for recovery between groups over time. The intervention group did show a slight improvement of the satisfaction of the need for relatedness over time, while in contrast, the control group showed a decrease over time. The satisfaction of the need for autonomy and competence did not significantly differ between both groups over time.

**Conclusions:**

The approach had no significant effect on the primary outcome need for recovery. The intervention did have a significant positive impact on the satisfaction of the need for relatedness, possibly because, after a period of being unable to be close, it provided opportunity to gather and work together as a team.

By 2050, the percentage of Europeans that are ≥80 years is expected to have doubled compared to 2015, indicating a growing demand for long-term care (1). This demand is further increased by high turnover rates in the long-term care profession, caused by challenging working conditions (1). With this expanding shortage of long-term care workers, it is necessary to improve long-term care workers’ ability and willingness to remain working in this sector. In other words; investing in these employees’ sustainable employability is essential (2). In order to remain working, a healthy work environment is key (3). Long-term care workers report that job demands need to diminish (eg, workload, rules and regulations) and job resources should be fostered (eg, autonomy, appreciation and development opportunities) (4). Experiencing a substantial amount of job demands in combination with limited resources not only drains energy, but also prevents workers from meeting their basic psychological needs (5). According to the Self-Determination Theory, individuals can only achieve their full potential when three basic psychological needs are met, namely the needs for autonomy, competence, and relatedness (6). People need to feel like they (i) act from their own personal interest and values (autonomy), (ii) are effective in what they do while being able to use their capabilities (competence), and (iii) have meaningful connections and belonging to others (relatedness) (7). Being fulfilled in these three basic needs relates to a variety of beneficial outcomes for employees that are in favor of employees’ sustainable employability (eg, health, vitality) (5, 8–10).

In the context of long-term care, teams consist of healthcare professionals who are jointly responsible for providing care and assistance for individuals, often elderly, who may be physically and/or cognitively impaired. Organizations in this setting increasingly adopt self-managing or -directing team structures. Self-managing teams are characterized by their ability to not only provide care and pay attention to the wellbeing of the residents but also perform tasks such as scheduling, performance monitoring and professional development without a supervisor or team leader (1). In order to foster sustainable employability of long-term care staff in organizations with self-managing teams, it therefore seems promising to focus on the team in forthcoming workplace interventions rather than on the individual (11–13).

Another promising element for interventions that aim to improve the work environment is employing a participatory approach (14). The participatory approach from Huysmans and colleagues (15) is an established intervention method designed to enhance workplace health and safety whereby a series of process steps are taken under the guidance of a facilitator. These steps pinpoint crucial bottlenecks at work, generate appropriate solutions using a concrete action plan, incorporating equal input from all stakeholders (15). Since steps to be taken need to be agreed upon, there is an increased likelihood that the newly implemented working methods will sustain over time (16, 17). In the context of self-management, where teams have diverse ways of working and associated challenges, the participatory approach adds to its promise by providing the opportunity to deal with this diversity (11).

Results from our qualitative needs assessment showed that it was considered important by employees themselves to address the needs for autonomy, relatedness and competence in order to remain willing and able to work (18). They indicated a need to strengthen control and involvement in decisions (need for autonomy), opportunities and support in development on a personal and team-level (need for competence) and a need for togetherness, support, and more attention to the residents (need for relatedness). Appreciation, in terms of finances, positive rewards, and feeling heard and seen were very important with regard to relatedness (18). Working conditions could be hindering in relation to sustainable employability, mostly by their ability to hinder the fulfilment of their needs for autonomy, relatedness and competence. For example, staff shortages and high workload hindered staff’s ability to spend time with residents (and thereby their need for relatedness) or impeded their team’s developmental progress (and thereby their need for competence) (18).

We therefore developed an intervention consisting of a participatory workplace approach at team level, further referred to as the Healthy Working Approach. This is one of the first studies on the participatory approach focused on self-managing teams, allowing teams to identify the most important bottlenecks at work, come up with appropriate solutions in a concrete plan of action and evaluate the steps. The rationale behind the approach was that by addressing bottlenecks, participants would be more satisfied in their needs for autonomy, relatedness and competence. This, in turn, should be beneficial to employees' sustainable employability. Sustainable employability is a broad concept that concerns having the opportunities and conditions to work, while being physically and mentally healthy, now and in the future (19). With sustainable employability being a complex multi-indicator construct, covering many domains, it is suggested to use indicators in longitudinal research to capture (changing) sustainable employability among individuals (20). Need for recovery, also known as post-work fatigue, is seen as one of those indicators (20). Since recovery requires the restoration of energy according to the effort–recovery model (21) and research based on Self-Determination Theory shows that meeting the needs for autonomy, relatedness and competence is related to energy preservation or enhancement (22), meeting the three needs is expected to facilitate recovery. This relationship is quantified by previous research showing that satisfaction of these needs is related to improved recovery during free evenings after work (23, 24) or on non-work days (25). The relationship is further supported by qualitative research with the target group, where long-term care workers express that the satisfaction of these needs is important for their sustainable employability (18). The aim of the Healthy Working Approach is therefore to improve need for recovery as indicator of sustainable employability of long-term care workers through improving fulfilment of their basic psychological needs at work.

To assess the effectiveness of the Healthy Working Approach, we have conducted a randomized controlled trial. Our primary focus in this evaluation was on employees’ need for recovery given its recognition as an indicator of sustainable employability, as a precursor to negative health issues and the well-being of employees in general (17–20) and due to its susceptibility to change (26). Additionally, we examined the impact of the intervention on the satisfaction of the needs for autonomy, competence, and relatedness. Our study aimed to address two key questions: (i) what is the effect of the Healthy Working Approach on the need for recovery of long-term care workers working in self-managing teams over a one year follow-up? and (ii) what are the effects of the Healthy Working Approach on the satisfaction of the needs for autonomy, competence and relatedness of long-term care workers working in self-managing teams over a one year follow-up?

## Methods

### Trial design

We conducted a randomized controlled trial with a waitlist control group in an intervention design. The study protocol was approved by the Ethical Committee Social Sciences of the Radboud University (number: ECSW-2021-012). The trial was registered in the Netherlands Trial Register (NL9627).

### Setting

There were two Dutch long-term care organizations involved in the study. Both organizations worked with self-managing teams and were mid-to-large sized with around 700 employees each.

### Participants and procedure

All employees were invited to participate in this study by information and videoclips on intranet, in physical meetings and presentations, by leaflets in shared areas and by e-mail. Long-term care workers could participate in the study if they were able to read and understand the Dutch language and were ≥18 years old. They were excluded from the study if they were on sick leave for ≥1 months or their employment contract would end within six months after completing the baseline questionnaire. Because of the team-level intervention, additional team-level inclusion criteria were applied. Teams were included in the study if at least one third of the team members completed the baseline questionnaire and ≥3 team members were willing to represent their team in the three meetings of the intervention (ie, take part in the working group).

Questionnaires were administered at baseline (T0), after 6 months (T6), 9 months (T9) and 12 months (T12). The baseline questionnaire started with an eligibility check according to in- and exclusion criteria followed by a digital informed consent. Data collection started in May 2021 and lasted until August 2023. To increase the response rate on the follow-up questionnaires, the researchers visited teams to bring over hardcopy questionnaires and provided incentives to teams that achieved a 75% response rate on the follow-up measurements (eg, a plant or photo frames).

### Allocation of intervention and waitlist control group

A research assistant with no knowledge of the teams performed pairwise team-level randomization using randomizer.org. The first author provided the research assistant with pairs of teams, aiming for similarities with regards to the kind of residents they served (ie, psychogeriatric, somatic or hospice) and team size (ie, around 10 or 20). After the completion of the 12-month follow-up questionnaires, the waitlist control group received a notification that they were also eligible to commence the intervention.

### Intervention: The Healthy Working Approach

A detailed description of the intervention alongside more information about the procedures and participant involvement, can be found in the study protocol related to this article (27). For each team, the intervention consisted of a problem assessment (meeting 1), related to the needs for autonomy, competence and relatedness, a brainstorm towards solutions and an action plan (meeting 2) and an evaluation (meeting 3). The meetings were held over a 3–6 month period and guided by a facilitator who was a trained employee from within the long-term care organization. Each team selected representatives to join a working group who would attend these meetings. The working group represented the entire team and therefore had to report back to the team after each meeting. The approach aimed to result in improvements that would benefit the whole team. To illustrate, one team identified communication as a bottleneck for their team and saw providing feedback as solution. In the action plan they agreed upon providing each other with feedback during each shift. In another team, differential work structures and subsequent expectations, were experienced as hurdles. Renewed agreements surrounding working methods were outlined in the onboarding document for new employees, according to the action plan.

### Outcome measures

The *primary outcome need for recovery* was measured with 11 dichotomous items (0 no or 1 yes) of the Need for Recovery Scale (28). An example item is *“I find it difficult to relax at the end of a workday”*. The need for recovery score is a percentage (0–100) of positive answers to the items, with a higher percentage indicating a higher degree of need for recovery after work. The need for recovery scale shows good (content) validity and internal consistency (26). Here Cronbach’s alpha was 0.82.

*Secondary outcomes satisfaction of the needs for autonomy, competence and relatedness* were measured with 16 items of the validated Work-related Basic Need Satisfaction Scale on a 5-point scale (ranging from 1 totally disagree to 5 totally agree) (29). Autonomy satisfaction was measured with 6 items, for example *“There is not much opportunity for me to decide for myself how to go about my work”* (reversed coded). An example of one of the 4 items measuring competence satisfaction is *“People at work tell me I am good at what I do.”* Six items measured relatedness satisfaction, for example *“I consider the people I work with to be my friends”*. Higher sum scores for the subscales autonomy, competence and relatedness, indicate a higher degree of satisfaction of the need at hand. The Work-related Basic Need Satisfaction Scale has been validated, and the subscales for autonomy, competence and relatedness satisfaction show good reliabilities (29). Cronbach’s alpha’s were 0.76 for autonomy, 0.86 for competence and 0.73 for relatedness.

### Confounding factors

At baseline, information about several confounding factors was collected, namely: age stratum (18–24, 25–54, ≥55 years), gender (male, female, other), educational level (elementary school, lower secondary education, upper secondary education, higher professional education, university education), profession (job titles based on three categories: care and well-being professionals, physicians and practitioners and support services personnel), years employed (<1, 1–5, 6–10, >10 years) and type of contract (permanent, temporary with prospect of permanent, temporary with fixed-term, temporary/on-call employee/freelancer), number of contractual working hours per week (<12, 12–20, 20–28, 28–35, >35 hours), hours of informal care provision per week in the last six months and frequency and total number of working days of sickness absence in the past six months.

### Sample size calculation

Prior to commencing data collection, a sample size calculation was performed by means of a t-test computation in G-Power 3.1 (30). The calculation was based on mean scores on need for recovery of employees in occupational health services, an 80% power and a 5% alpha and resulted in the need for 202 long-term care workers in our study (see study protocol for details; 27). Due to the COVID-19 pandemic and its consequences on long-term care, the process of recruiting teams for our study proved to be more challenging and time-consuming than expected. To ensure a more recent and appropriate sample size, we recalculated the required number of participants in January 2022, using baseline data collected at that time on need for recovery (N=105). Since interventions with a similar approach were able to cause changes in the work environment, that in turn lead to changes in burnout (31), it is likely to find an effect on need for recovery (the precedent of burnout). Considering a minimally relevant difference of 12 points (26, 32), using the ICC for teams at 0.025, 80% power and 5% alpha, the recalculation indicated a need for 106 participants. Factoring in a 25% attrition rate, the final sample size showed that 142 long-term care workers were needed (71 in the intervention group and 71 in the control group).

### Statistical methods

We calculated descriptive statistics (frequencies, means, and standard deviations) on all outcomes and confounding factors. To test effects and control for the non-independence of the nested data (individuals in teams over time), a mixed-models analysis was run in R using lme4-package (33). Before analyzing the data, a data analysis plan was preregistered, (osf.io/kuc4x/?view_only=4c4c03ce91ab4161a953227fc07f6f04). For the main analyses, we estimated a model for each dependent variable (need for recovery and satisfaction of the needs for autonomy, competence and relatedness) with fixed effects for time [continuous ranging from 0 (T0) to 3 (T12)], group (intervention versus control), and time×group, and random effects for participant and team. Following our preregistration, we checked whether the intervention and control group differed from each other on each of the potential confounders (P<0.15) at baseline. If this was the case, the potential confounders were added one-by-one to the main model, separately for each dependent variable (need for recovery and satisfaction of the needs for autonomy, competence and relatedness). If adding the confounder to the main model resulted in at least 10 percent change in the size of the interaction term (time×group), the confounder was retained in the model. Main analyses were performed according to the “intention to treat” principle, meaning we used the allocated group status into account (control group = 0, intervention group = 1). As a secondary analysis we performed a per-protocol analysis in which we compared teams that had the intended three meetings (indicated by 1) with the teams with <3 meetings, including the waitlist control teams (indicated by 0). To compute P-values, we estimated degrees of freedom using Kenward Roger approximation.

## Results

### Description of participants

We randomized 20 teams including 136 participants in this study. The flow diagram (figure 1) shows that 82 long-term care employees who completed the baseline questionnaire, did not meet the inclusion criteria. The majority of the participants came from organization 1 (N=108) in comparison to organization 2 (N=28). Ten teams were randomized into the intervention group (N=78) and ten teams into control group (N=58). Of the intervention group, seven teams (56 participants) had received all three intended meetings of the intervention. Two teams did not have any meetings and one team only held the first meeting. During the follow-up measurements response rates varied, but across all time points the overall response rate for the primary outcome measure was 79%.

**Figure:**
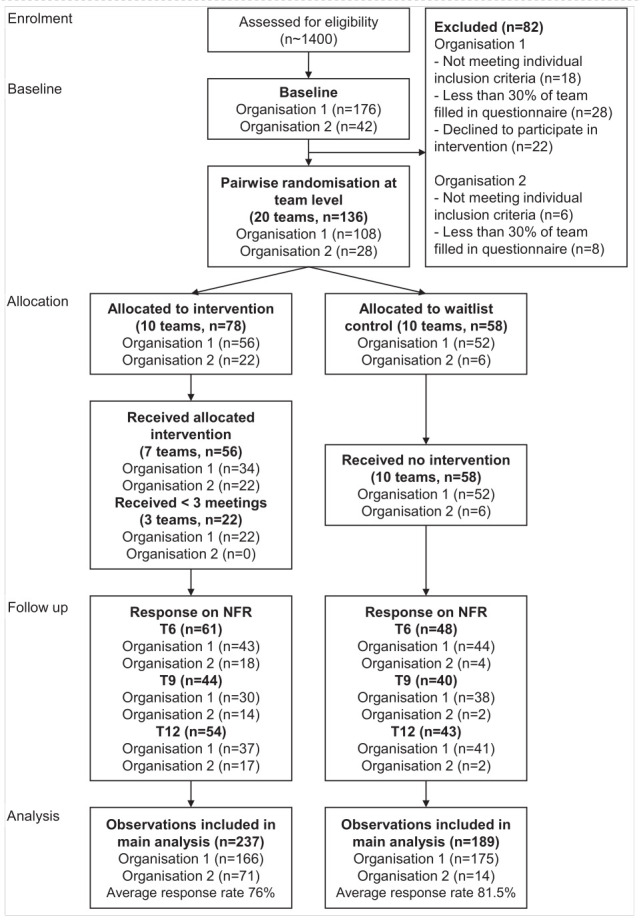
**Figure 1.** Flow diagram of participants through the study.

Table 1 presents participant characteristics. Most participants were aged 18–54 years, female, and had completed higher secondary education. The majority of participants worked >20 hours per week, around 40% worked >28 hours per week. Almost half of the participants worked >10 years in long-term care. Participants in the control group spent on average 7.3 hours per week on informal caregiving, in the intervention group this was 5.8 hours per week. The intervention group differed from the control group (P<0.15) regarding profession, type of contract and frequency and total number of working days of sickness absence. Compared to the control group, the intervention group consisted of more care and well-being professionals (88.5% versus 72.4%), less employees in support services (3.8% versus 19.0%), had less often permanent contracts (79.5% versus 89.7%) and reported a higher frequency and duration of sickness absence in the past month (1.5 versus 0.4 day).

**Table 1:** Mean, standard deviation (SD) and row percentages of participant (N) characteristics.

Characteristics		Intervention group (N=78)					Control group (N=58)			
		N	%	Mean	SD		N	%	Mean	SD
Age in years										
	18–54	56	71.8				40	69.0		
	≥55	22	28.2				18	31.0		
Gender										
	Male	8	10.3				4	6.9		
	Female	70	89.7				54	93.1		
	Else	0	0.0				0	0.0		
Educational level										
	Low	17	21.8				9	15.5		
	Intermediate	36	46.2				25	43.1		
	High	25	32.1				24	41.4		
Profession **										
	Care and well-being professionals	69	88.5				42	72.4		
	Physicians and practitioners	6	7.7				5	8.6		
	Support services personnel	3	3.8				11	19.0		
Years employed										
	<1	11	14.3				10	17.2		
	1–10	24	31.2				24	41.4		
	>10	42	54.5				24	41.4		
Type of contract *										
	Permanent contract	62	79.5				52	89.7		
	Temporary contract	16	20.5				6	10.3		
Contractual working hours per week										
	<20	9	11.5				7	12.1		
	20–28	36	46.2				28	48.3		
	>28	33	42.3				23	39.7		
Hours of informal care provision per week				5.76	17.42				7.29	22.52
Frequency sickness absence in past month **				0.30	0.75				0.11	0.32
Working days of sickness absence in past month *				1.59	3.99				0.48	1.32

* Significant difference (P<0.15) between intervention versus control group. ** Significant difference (P<0.05) between intervention versus control group.

### Results of analysis for need for recovery

The main analysis revealed no significant difference in need for recovery between groups over time (table 2). However, there was a significant positive main effect of time on need for recovery, which indicated that need for recovery increased over time for both groups [estimate = 3.8, standard error (SE) = 1.2, t (14) = 3.2, P<0.01]. For the intervention group, need for recovery increased from an average of 35.69 at baseline to 45.62 at the 12-month follow-up, while the control group increased from 28.47 to 38.05 in the same time frame (table 3).

**Table 2:** Estimates, standard error (SE), degrees of freedom (Df) and t-values of fixed effects with number of observations in the model (N) according to intention to treat (control group=0, intervention group=1).

Fixed effects	Need for recovery ^1^ (N=426)					Need for autonomy ^2, 3^ (N=410)					Need for competence ^1^ (N=414)					Need for relatedness ^1^ (N=414)			
	Estimate	SE	Df	t-value		Estimate	SE	Df	t-value		Estimate	SE	Df	t-value		Estimate	SE	Df	t-value
Intercept	29.63	4.33	18.98	6.83 ***		22.61	0.45	19.21	49.79 ***		16.57	0.34	19.01	49.14 ***		23.76	0.46	18.96	51.91 ***
Time	3.80	1.19	13.99	3.20 **		-0.28	0.21	17.20	-1.33		-0.14	0.12	14.84	-1.21		-0.73	0.19	15.70	-3.80 **
Group	4.97	5.97	16.08	0.83		1.31	0.61	14.72	2.14 *		-0.26	0.46	15.87	-0.56		-0.73	0.62	14.84	-1.18
Time×group	-1.14	1.59	12.38	-0.72		0.13	0.28	15.05	0.45		0.22	0.15	12.80	1.45		0.92	0.25	13.55	3.62 **

^1^ Optimizer bobyqa used in model. ^2^ Optimizer nmkbw used in model. ^3^ Model adjusted for variable “frequency of sickness absence”. *** P<0.001. ** P<0.01. * P<0.05

**Table 3:** Observed means, standard deviations (SD) of participants (N) of primary and secondary outcomes categorized by group (intervention and control) and time (T0, T6, T9, T12).

		Time														
		T0				T6				T9				T12		
Outcome	Group	Mean	SD	N		Mean	SD	N		Mean	SD	N		Mean	SD	N
Need for recovery	Control	28.47	24.38	58		33.71	28.81	48		31.36	25.63	40		38.05	30.30	43
	Intervention	35.69	28.26	78		37.85	28.18	61		44.83	29.82	44		45.62	30.51	54
Need for autonomy	Control	22.74	3.27	57		22.15	4.34	47		22.90	4.09	40		22.31	4.08	36
	Intervention	23.83	2.89	77		23.49	3.21	61		23.73	3.41	44		23.13	3.27	52
Need for competence	Control	16.72	1.81	57		16.13	2.63	47		16.65	2.46	40		16.39	2.28	36
	Intervention	16.47	2.31	77		15.84	2.15	61		16.70	2.01	44		16.29	2.37	52
Need for relatedness	Control	24.05	2.92	57		22.49	3.78	47		22.78	3.29	40		22.28	3.95	36
	Intervention	23.06	3.60	77		23.07	3.11	61		23.00	3.59	44		23.37	3.58	52

### Results of need for autonomy, competence and relatedness

In the main analysis (table 2), a significant positive time×group interaction effect was found for relatedness satisfaction [estimate = 0.9, SE = 0.3, t (14) = 3.6, P<0.01]. This interaction implies that relatedness satisfaction decreased for the control group at a rate of 0.73 per time-point, but increased for the intervention group at a rate of 0.19 over time-points (not shown). The intervention group averaged in satisfaction of relatedness from of 23.06 at baseline to 23.37 at the 12-month follow-up, while the control group decreased in their fulfilment from 24.05 at baseline to 22.28 at the 12-month follow-up (table 3). In general, there is a decrease in the satisfaction of the need for relatedness over time, indicated by a significant negative main effect of time on relatedness satisfaction [estimate = -0.7, SE = 0.2, t (16) = -3.8, P<0.01]. No significant effects of time×group were found for both autonomy and competence satisfaction. For autonomy there was a significant group effect, indicating that the intervention group expressed higher levels of autonomy satisfaction [estimate = 1.3, SE = 0.6, t (15) = 2.1, P<0.05]. Across all time-points, levels of autonomy satisfaction were on average about one point higher in the intervention group (eg, at baseline: 23.83 versus 22.74) (table 3).

### Results of per protocol analyses

The results from the secondary per protocol analyses (table 4) substantiate the findings of the main analyses. No significant effects of time×group were found for need for recovery, autonomy and competence. This indicates that there are no significant differences between teams with three versus less than three meetings over time in the per protocol analyses for these outcomes. Similar to the main analyses, a significant positive team per protocol×time interaction effect was found for relatedness satisfaction [estimate = 0.7, SE = 0.3, t (14) = 2.1, P<0.05]. This interaction reveals that while relatedness satisfaction decreased for teams with less than three meetings at a rate of 0.51 per time point, it increased for teams with three meetings at a rate of 0.15 over time-points (not shown).

**Table 4:** Estimates, standard error (SE), degrees of freedom (Df) and t-values of fixed effects with number of observations in the model (n) according to team per protocol (teams with less than 3 meetings=0, teams with 3 meetings=1).

Fixed effects	Need for recovery ^1^ (N=426)					Need for autonomy ^2^ (N=414)					Need for competence ^1^ (N=414)					Need for relatedness ^2^ (N=414)			
	Estimate	SE	Df	t-value		Estimate	SE	Df	t-value		Estimate	SE	Df	t-value		Estimate	SE	Df	t-value
Intercept	29.31	3.83	17.25	7.66 ***		22.89	0.40	14.94	56.81 ***		16.55	0.30	16.88	55.60 ***		23.83	0.37	13.20	64.37 ***
Time	3.55	1.05	13.45	3.37 **		-0.35	0.18	17.31	-1.91		- 0.08	0.10	15.11	-0.72		-0.51	0.20	17.84	- 2.52 *
Team per protocol	7.55	6.06	14.85	1.25		1.00	0.63	13.06	1.59		-0.31	0.47	14.54	-0.66		-1.25	0.57	11.74	-2.19 *
Time * Team per protocol	-0.89	1.59	11.46	-0.56		0.31	0.28	13.70	1.12		0.13	0.15	11.67	0.86		0.66	0.31	14.21	2.15 *

^1^ Optimizer bobyqa used in model. ^2^ Optimizer nmkbw used in model. *** P<0.001. ** P<0.01. * P<0.05.

## Discussion

The main objective of this study was to acquire insight into the effectiveness of the Healthy Working Approach on the primary outcome need for recovery and secondary outcomes satisfaction of the needs for autonomy, relatedness and competence among long-term care workers working with self-managing teams over a one-year period. The results of this study showed no indications of a significant effect of the Healthy Working Approach on the primary outcome need for recovery. The approach did have a significant positive effect on the satisfaction of the need for relatedness. No significant effects were found for the satisfaction of the needs for autonomy nor competence.

### Comparison with other studies and interpretation of results

In our study, an effect of the Healthy Working Approach on need for recovery remained absent. The previously found positive results of participatory approaches on health outcomes in health care settings (eg, hand eczema, backpain or burnout) do not resonate with current findings (31, 34–37). This discrepancy may be attributed to the fact that need for recovery is a less specific and more distal outcome compared to measures like hand eczema and back complaints. Notably, proximal outcomes more often show positive results than distal outcomes (31, 38). The causal chain in the intervention is considerable in length and involved addressing unfavorable working conditions on team-level in order to enhance the fulfilment of the basic psychological needs at work, thereby decreasing need for recovery. From current evaluation, it is unclear to what extent working conditions actually changed and whether this had the presumed follow-up effects. We furthermore cannot conclude about the effects of specific actions, as all teams chose their own actions based on the team’s bottlenecks. However, in line with previous studies applying the participatory approach (31, 34–37), the effective mechanism is the process of systematically identifying bottlenecks and solutions that match the teams’ needs. It is furthermore important for interventions in the work context that enough employees are involved in the implementation activities (31). With only a representation of each team involved in the workgroup of the intervention and a large part of our sample working part-time (mostly 20–28 hours per week), it is possible that the employees in the intervention group are not sufficiently exposed to or engaged in the (implementation of) solutions. However, including more team members in the workgroups creates challenges with regards to planning the meetings. Another reason for the absence of an effect on need for recovery might be that this study took place in (the aftermath) of the COVID-19 pandemic in The Netherlands, of which we know it added pressure on long-term care staff with increased workloads from new infection control measures, higher sickness absence of colleagues, which in addition increased workload and stress of becoming infected or infecting others (39). In our sample, means on need for recovery were 28.47 for the control group and 35.69 for intervention group at baseline. This increased to 38.05 for the control group and to 45.62 for the intervention group at the 12-month follow-up. Of the observations at the 12 month-follow up, 66% of the participants were considered to have a high need for recovery (score >54.5). In comparison, this is 19% for hospital care nurses and 30% for home care staff (40). Assuming consequences due to the COVID-19 pandemic had an impact on the development of need for recovery over time, there might have been limited space for the intervention to be effective in terms of need for recovery. A last remark on the lack of effect on need for recovery has to do with possible self-selection in our study. In order to be included in the trial at least 30% of a team had to complete the baseline questionnaire. This threshold, along with the motivation and time-effort needed to take part in the intervention, may have resulted in a selective sample. Although we have no information on those who declined participation, it was emphasized during recruitment that even though participating was a time investment, it was a way to tackle current issues (and therefore likely to be beneficial long-term). Some teams in our sample did not have all the intended three meetings, which indicates that not only the more motivated and fortunate staffed teams joined our trial. Since scores on need for recovery at baseline can be considered average, it is possible that the teams that would benefit most from the intervention, did not participate in the study.

To our knowledge, self-determination theory has not been applied within the context of a participatory team-level intervention. Despite this, it is promising to see that such an intervention has a positive impact on the satisfaction of the need for relatedness of long-term care workers. It is argued that fulfilling the need for relatedness, creates an intrinsically motivating atmosphere, which leads to higher sustainable employability (41). Other studies conducted in the same period showed that the pandemic intensified the need for collaboration (25, 26) and due to social distance restrictions and changing teams, it was harder to maintain a good team climate (42). The process steps of the intervention might therefore have been especially fitting with employees’ need for relatedness that, after a period of not being able to be close, provided the opportunity of coming and working together as a team. The intervention did not show an effect on the satisfaction of the need for autonomy and competence. Since the intervention was on a team-level, personal barriers or other needs may not have been addressed. In other words, what a team needs (and acts on) will hopefully contribute to individual need fulfillment but will likely not overcome all individual issues as these might require an individual-level intervention. It is furthermore possible that in the intervention steps, team members focused on bottlenecks relating to relatedness rather than autonomy and competence. Consequently, the intervention may not have adequately addressed the specific challenges related to the needs for autonomy and competence within the long-term care teams to be effective in meeting these needs.

### Strengths and limitations

Despite recalculating the required sample size during data collection, our study fell short of the initially estimated sample for the control group. The final sample size was determined to consist of 142 long-term care workers (71 each in the intervention and control groups). We randomized 136 long-term care workers (78 in the intervention and 58 in the control group). Furthermore, our study design does not provide precise insight into what actions in teams entailed and what their short/long-term effects were because measurements in this study were not related to the meetings/and or actions itself. Meetings and subsequent actions could have taken place long- or shortly before or after a certain measurement. In addition, not all team members attended the meetings, which could have limited the support for solutions and therefore the effectiveness of the approach. However, this is one of the first studies investigating the impact of the participatory approach focusing on self-managing teams with a basis in self-determination theory. The thoroughness of the pre-registered data analysis is one of the assets of this study. In this, we took into account the nestedness and missings info in the data and applied intention-to-treat and per protocol analyses to better understand the effectiveness of the intervention. The longitudinal repeated measures design, including a control condition, further strengthens our study.

### Recommendations for future research

The increase in need for recovery over time, indicates a need to remain focused on reducing and preventing the possible results from long-term post-work fatigue. Since we found an impact of the Healthy Working Approach on the satisfaction of relatedness during described circumstances (during the COVID-19 pandemic and with a smaller control group), there is some benefit to the intervention. A replication of the study, with a sufficient sample size and the inclusion of teams high on need for recovery could provide more insight into the effect of such an workplace participatory intervention. Currently, it is unknown how the intervention was exactly implemented and how this implementation process might have affected the results. The accompanying process evaluation of this trial will therefore offer the opportunity to assess whether the impact of the approach on relatedness – in contrast to need for recovery, autonomy and competence – was indeed the result of a prioritization or appreciation of this element by the workgroup members in the intervention. A better understanding of what happened during the intervention can place present results more accurately into context.

### Concluding remarks

With the current randomized controlled trial we evaluated the effectiveness of the Healthy Working Approach, an intervention consisting of a participatory workplace approach at team level, on long-term care workers in self-managing teams. Results showed that the approach had no significant effect on the primary outcome need for recovery. The approach did have a significant positive impact on the satisfaction of the need for relatedness. The intervention group slightly improved in their satisfaction of the need for relatedness over time, while the control group showed a decrease over time. No significant results were found for the other two secondary outcomes: the need for autonomy and competence. Future research could benefit from using a sufficiently large sample, including teams high in need for recovery, and a process evaluation to provide more insight into the value of current results.

## Data Availability

Data and scripts are available upon request from: https://doi.org/10.34973/5fsr-r060.
